# The planetary biology of cytochrome P450 aromatases

**DOI:** 10.1186/1741-7007-2-19

**Published:** 2004-08-17

**Authors:** Eric A Gaucher, Logan G Graddy, Tang Li, Rosalia CM Simmen, Frank A Simmen, David R Schreiber, David A Liberles, Christine M Janis, Steven A Benner

**Affiliations:** 1 Foundation for Applied Molecular Evolution, 1115 NW 4th Street, Gainesville FL 32601-4256, USA; 2 Department of Psychiatry, Duke University Medical Center, Durham, NC 27708, USA; 3 Department of Physiology & Biophysics, Medical Sciences & Children's Nutrition Center, University of Arkansas, 1120 Marshall Street, Little Rock AR, 72202, USA; 4 Computational Biology Unit, Bergen Center for Computational Science, University of Bergen, 5020 Bergen, Norway; 5 Ecology and Evolutionary Biology, Brown University, Providence RI 02912, USA; 6 Department of Chemistry, University of Florida, Gainesville FL 32611-7200, USA

## Abstract

**Background:**

Joining a model for the molecular evolution of a protein family to the paleontological and geological records (geobiology), and then to the chemical structures of substrates, products, and protein folds, is emerging as a broad strategy for generating hypotheses concerning function in a post-genomic world. This strategy expands systems biology to a planetary context, necessary for a notion of fitness to underlie (as it must) any discussion of function within a biomolecular system.

**Results:**

Here, we report an example of such an expansion, where tools from planetary biology were used to analyze three genes from the pig *Sus scrofa *that encode cytochrome P450 aromatases–enzymes that convert androgens into estrogens. The evolutionary history of the vertebrate aromatase gene family was reconstructed. Transition redundant exchange silent substitution metrics were used to interpolate dates for the divergence of family members, the paleontological record was consulted to identify changes in physiology that correlated in time with the change in molecular behavior, and new aromatase sequences from peccary were obtained. Metrics that detect changing function in proteins were then applied, including K_A_/K_S _values and those that exploit structural biology. These identified specific amino acid replacements that were associated with changing substrate and product specificity during the time of presumed adaptive change. The combined analysis suggests that aromatase paralogs arose in pigs as a result of selection for Suoidea with larger litters than their ancestors, and permitted the Suoidea to survive the global climatic trauma that began in the Eocene.

**Conclusions:**

This combination of bioinformatics analysis, molecular evolution, paleontology, cladistics, global climatology, structural biology, and organic chemistry serves as a paradigm in planetary biology. As the geological, paleontological, and genomic records improve, this approach should become widely useful to make systems biology statements about high-level function for biomolecular systems.

## Background

The emergence of complete genomes for many organisms, including humans, has created the need for hypotheses concerning the "function" of specific genes that encode specific proteins. While "function" is interpreted by different workers in different ways [1], Darwinian theory (by axiom) requires that the term be connected to fitness; natural selection is the only mechanism admitted by theory to generate functional behavior in a living system, macro or molecular. This, in turn, implies that the hypotheses about function have a "systems" component, including the interaction of the protein with other proteins, their impact on the physiology (defined broadly) of the cell and organism, and the consequences of physiology in a changing ecosystem in a planetary context [2].

Systems hypotheses can be supported by information from many areas. Geology, paleontology, and genomics, for example, provide three records that capture the natural history of past life on Earth. At the same time, structural biology, genetics, and organic chemistry describe the structures, behaviors and reactivities of proteins that allow them to support present life. It has been appreciated that a combination of these six types of analysis provides insights into functional behavior of proteins that cannot be provided by any of these alone [2]. Over the long term, we expect that the histories of the geosphere, the biosphere, and the genosphere will converge to give a coherent picture showing the relationship between life and the planet that supports it. This picture will be based, however, on individual cases that serve as paradigms for making the connection.

The aromatase family of proteins offers an interesting system to illustrate the power of this combination as a way to create hypotheses regarding protein function within a system [3]. These hypotheses are not "proof", of course, but are limiting in genomics-inspired biological experimentation, now that genomic data themselves are so abundant.

**Figure 1:**
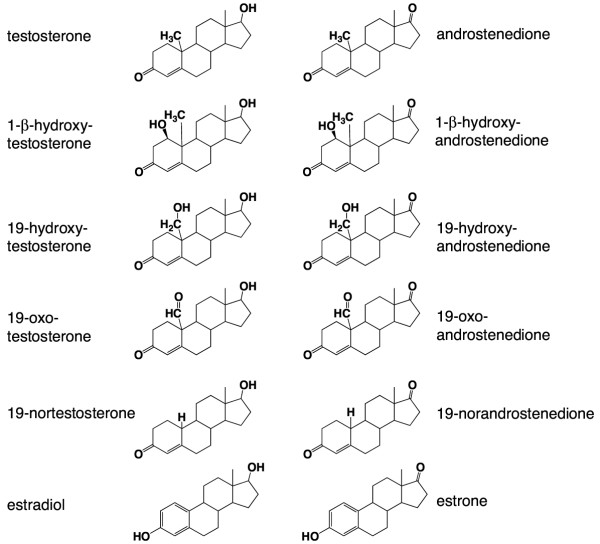
Reactions catalyzed by aromatases on multiple androgenic substrates.

Aromatases are cytochrome P450-dependent enzymes that use dioxygen to catalyze a multistep transformation of an androgenic steroid (such as testosterone) to an estrogenic steroid (such as estradiol) (Figure 1). The protein plays a key role in normal vertebrate reproductive biology–a role that appears to have arisen before fish and tetrapods (land vertebrates, including mammals) diverged some 375 million years ago [4]. Aromatase is important in modern medicine as well, especially in breast and other hormone-dependent cancers [5].

Different numbers of aromatase genes are found in different vertebrates. Two aromatase genes are known in teleost fish [6,7]. Only a single gene is known in the horse [8], rat [9], and mouse [10]. Cattle have both a functional gene and a pseudogene built from homologs of exons 2, 3, 5, 8, and 9 of their functional gene; these are interspersed with a bovine repeat element [11,12]. In several mammalian species, including humans and rabbits, a single gene yields multiple forms of the mRNA for aromatase in different tissues via alternative splicing [13-16].

A still different phenomenology is observed in the pig (*Sus scrofa*). Three different mRNA molecules had been reported in different tissues from pig [17-21]. Compelling evidence then emerged that the three variants of mRNA identified in cDNA studies arose from three paralogous genes [22], rather than from a single gene differentially spliced [23]. This implies that the three aromatase paralogs in pigs arose via gene duplications relatively recent in geologic time.

Hypotheses relating to the function of the three aromatase paralogs depend in part on when those duplications took place. If they were very recent, the three genes might have helped pigs adapt to domestication. If they pre-dated the divergence of pigs and fish [6], they may have different roles that are very fundamental to reproductive endocrinology in vertebrates. We apply here a series of tools to generate better hypotheses concerning the aromatase family of paralogs in swine.

## Results

One strategy useful for understanding the function of genes correlates events in their molecular evolution with events occurring in the history of other genes in the same and/or neighboring lineages, and with events recorded in the geological and paleontological records [2]. We incorporated a tool to date the divergence of two or more genes through an analysis of transitions at synonymous sites of two-fold redundant coding systems, where the encoded amino acid has been conserved [24]. This analysis exploits the approach-to-equilibrium kinetic behavior displayed by these sites. The analysis yields a transition redundant exchange (TREx) distance for any gene pair where the synonymous sites have not equilibrated.

To calibrate the silent TREx clock, inter-taxa histograms relating pig (*Sus scrofa*) and ox (*Bos taurus*) were constructed for transitions at the silent sites of two-fold redundant codon systems where the encoded amino acid was conserved between the species [24]. The major peaks associated with the separation of these two lineages was observed at *f*_2 _= 0.87, corresponding to a TREx distance of *kt *= 0.332. As the fossil record constrains the date of divergence of these two lineages to be 60 ± 5 Ma [25-27], and the codon biases in modern *Sus scrofa *and *Bos taurus *project an equilibrium value for *f*_2 _= 0.54 [24], the rate constants for transitions at the TREx silent sites were estimated to be ca. 2.8 × 10^-9 ^transitions/silent site/year during the time interval that separates these lineages.

**Figure 2:**
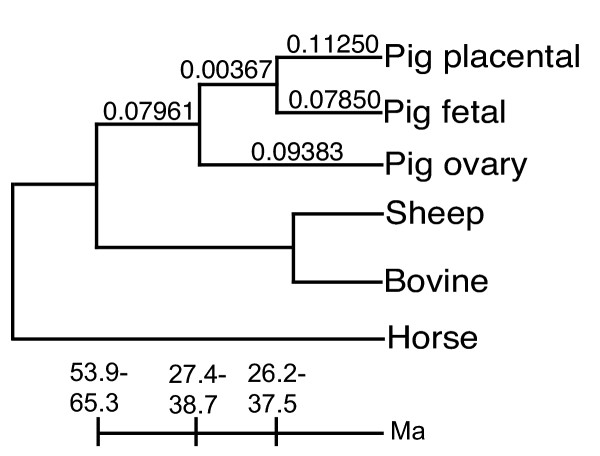
Dating the pig duplication events. An evolutionary tree, following the topology of Figure 5, showing estimated TREx distances for individual branches calculated from reconstructed ancestral sequences. The scale corresponds to evolutionary time (in million years) estimated from the TREx's using a first order rate constant for transitions of 3 × 10^-9 ^changes per base per year.

Analogous *f*_2 _values were then obtained for other vertebrate aromatase pairs, including fish *vs. *tetrapods (*f*_2 _= 0.56), birds versus mammals (*f*_2 _= 0.612), primates versus ungulates (*f*_2 _= 0.823), and horses versus artiodactyls (*f*_2 _= 0.828). Assuming a time-invariant single lineage first order rate constant of 3.6 × 10^-9 ^changes/site/year and an equilibrium *f*_2 _of 0.54, the corresponding dates of divergence are calculated to be 435, 258, 67, and 65 Ma respectively, with the oldest dates being the least precise. The last three of these dates of divergence are similar to those suggested by the paleontological record [28], within the error of the calculation, which reflects the modest number of characters used to calculate the *f*_2 _values. A tree for the artiodactyl lineage was constructed from the corresponding TREx distances (Figure 2). This was found to be consistent with the tree constructed from other metrics.

The TREx clock is not widely used. It may, however, provide more accurate dates in regions where synonymous transitions have not equilibrated than conventional clocks that combine data from synonymous transitions and synonymous transversions, or from non-synonymous changes. A comparison of different clocks will be provided in detail elsewhere (Benner *et al.*, in preparation). Briefly, the rate constants for transitions and transversions are more different than the two rate constants for purine-purine and pyrimidine-pyrimidine transitions. Further, nucleotide frequencies can be used to calibrate the end equilibrium points for two-fold redundant codon systems directly, and this permits an "approach to equilibrium" formalism, well known in chemical kinetics, to be applied [24,29-31].

From the tree, the TREx distances from the ancestor of fetal and placental aromatase to the modern enzymes are 0.113-0.079 (using an endpoint of 0.54 to reflect equilibration at the silent sites), corresponding to a range in the time of divergence of 26–38 Ma. The TREx distances from the divergence of all of the porcine aromatases and the modern forms ranges from 0.082–0.116, corresponding to dates of divergence in the range of 27–39 Ma. This suggests that the three aromatase paralogs diverged in the late Eocene to mid Oligocene.

**Figure 3:**
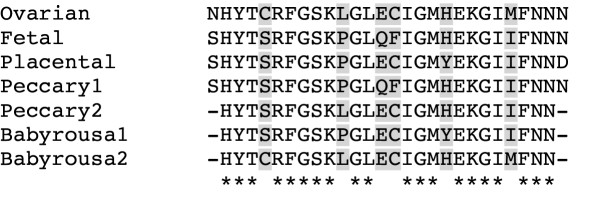
The amino acid alignment of exon 4 of two aromatase isoforms from both peccary and babirusa sequences with exon 4 of pig aromatase isoforms ovarian, fetal, and placental. Asterisks represent conserved sites.

To further correlate the duplication of the genes with the fossil record, genomic DNA was analyzed from relatives of *Sus scrofa*. Both peccary and babirusa seminal plasma (*Tayassu pecari*, from the Center for Reproduction of Endangered Species, Zoological Society of San Diego; *Babyrousa babyrussa*, from the Bronx Zoo, New York) was probed by PCR (Polymerase Chain Reaction) amplification using exon 4-specific primers [32]. Bands having the sizes expected for the corresponding aromatases were observed by agarose gel electrophoresis. Based on sequence similarity, two isoforms of aromatase were obtained from both peccary and babirusa as clones derived from the PCR products (Figure 3). This establishes that at least one of the duplications occurred before the Tayassuidae (the peccaries) diverged from the Suidae (the true pigs) ca. 35 Ma [33,34].

These data are consistent with an evolutionary model that holds that the ancestor of pig and oxen (approximated in the fossil record by *Diacodexis*, from the early Eocene ca. 55 Ma) [35] contained a single aromatase gene, and that the paralogous genes in pig arose some 20 million years later. This suggests that the paralogs in pig can be explained neither in terms of the fundamentals of vertebrate reproductive endocrinology, nor as a consequence of swine domestication.

This does, however, suggest that the emergence of the aromatase paralogs was approximately contemporaneous with the emergence of a litter in the Suoidea larger than that found in the ancestral artiodactyl condition. While ruminant and camelid artiodactyls have only one-two young per litter, suoids in general have at least two young per litter (as seen in peccaries) and most suines (true pigs) routinely have three-four young (up to 12 in the domestic pig, *Sus*). Note that there has long been the tacit assumption that large litters in suoids represent the primitive artiodactyl condition. Large litters are primitive for mammals in general, and because suoids are plesiomorphic in some anatomical conditions relative to other artiodactyls (e.g., short legs, retention of four digits, bunodont cheek teeth), they have been assumed to be plesiomorphic in other respects.

**Figure 4:**
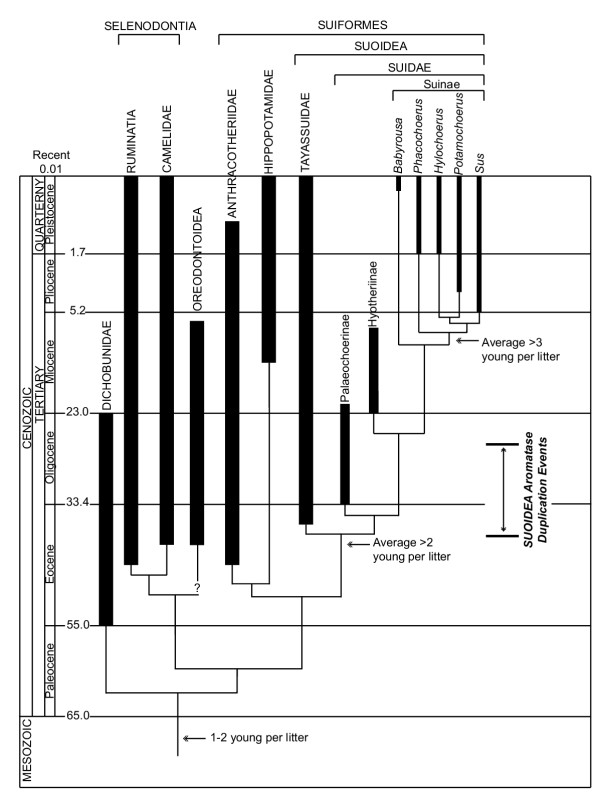
Cladogram of the order Artiodactyla showing the extant families and some selected extinct ones. Ruminantia includes the modern families Tragulidae, Giraffidae, Bovidae, Moschidae, and Cervidae, plus a number of extinct families. "Dichobunidae" is a paraphyletic assemblage of primitive taxa considered broadly ancestral to the later families. The interrelationships of the families reflect the "traditional" relationship based on morphology [85]. Different arrangements based on molecular information [86, 87] would alter the placement of the Camelidae and Hippopotamidae but would make no difference to the arguments presented here concerning the Suoidea. The interrelationships within the Suidae are based on information in several studies [32, 67, 88, 89]. Note that only a couple of extinct suid subfamilies are shown, and that only extant genera of Suinae are shown. Thick, medium-thick and thin lines represent family or above, subfamily and genera, respectively.

Other data suggest that small litters are in fact the primitive artiodactyl condition. Tragulids (mouse deer or chevrotains) are surviving small, primitive ruminants that are not too dissimilar from *Diacodexis *in body form, but only have one-two young per litter. Additionally, fossil record data on pregnant oreodonts (an extinct group probably related to the ruminant/camelid artiodactyl lineage, but with a suoid-like plesiomorphic postcranial morphology) shows that they also only had one-two young [36,37]. A cladogram of the Artiodactyla (Figure 4) illustrates the probable acquisition of multiparous versus uniparous reproductive strategies, and places the character of litters with typically more than two members emerging just before the divergence of Tayassuidae and Suidae.

The approximate correlation in time of the aromatase divergence in Suoidea with the enlargement of litters in Suoidea suggests, as a hypothesis, that the two are functionally related. To expand on this hypothesis, we sought genomic signatures of functional change within the aromatase paralogs. The number of non-synonymous changes in the gene divided by the number of the synonymous changes, normalized for the number of non-synonymous and synonymous sites (the K_A_/K_S _value), strongly suggests functional change when the value is significantly greater than unity [38,39], and is also an indicator of hypothetical functional change when the value is high on a branch of a tree relative to other branches of the same tree [40-43]. K_A_/K_S _values were reconstructed for individual branches of the evolutionary tree derived from the Darwin bioinformatics workbench (see Methods) using a distance matrix and ancestral states constructed by the method of Messier and Stewart [39]. The typical branch in the aromatase evolutionary tree has a K_A_/K_S _value of 0.35. A higher K_A_/K_S _value of 0.85 is found in the episodes of evolution near when the pig aromatases diverged. While a K_A_/K_S _value of 0.85 does not require the conclusion that positive selection occurred during the emergence of these aromatase paralogs, an inference based on the magnitude of K_A_/K_S _in one branch, relative to the K_A_/K_S _value for typical branches [40-43], suggests that adaptive changes occurred during the duplications of the aromatase genes in pigs.

**Figure 5:**
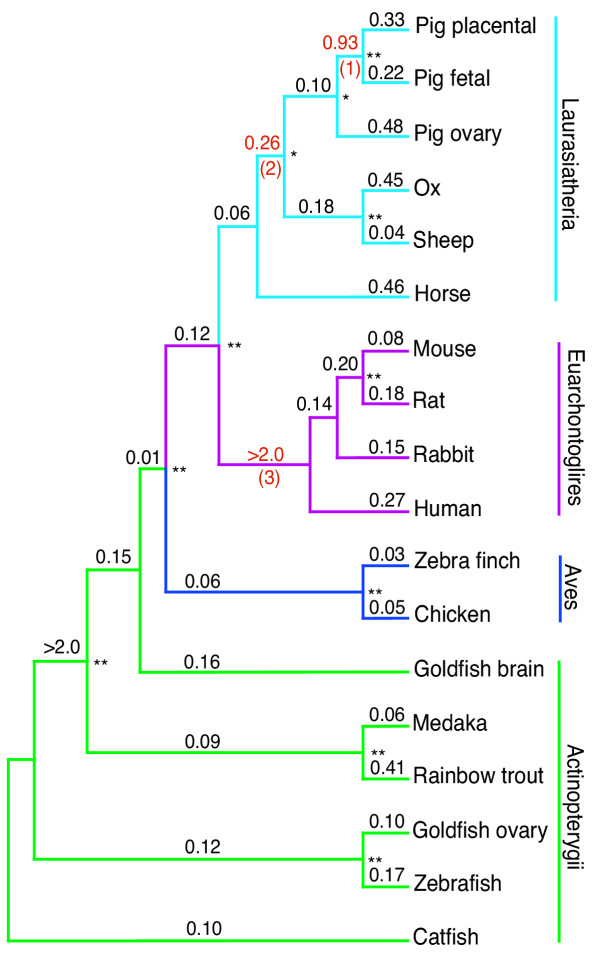
Phylogenetic tree for the 18 vertebrate aromatase genes. Numbers above the branches represent the K_A_/K_s _ratios, while numbers below indicate branches highlighted in Figure 6. Single and double asterisks represent bootstrap values of 95–99% and 100%, respectively. The following sequences were used: *Tilapia nilotica *(rainbow trout), gi:1613859, *Oryzias latipes *(medaka), gi:1786171, *Danio rerio *(zebrafish), gi:2306966, *Carassius auratus *(goldfish, ovary), gi:2662330, *Ictalurus punctatus *(catfish), gi:912802, *Carassius auratus *(goldfish, brain), gi:2662328, *Sus scrofa *(pig) placental, isoform 2, gi:1762232, *Sus scrofa *(pig) embryo, isoform 3, gi:1244543, *Sus scrofa *(pig) ovary, isoform 1, gi:1928957, *Bos taurus *(ox), gi:665546, *Equus caballus *(horse), gi:2921277, *Mus musculus *(mouse), gi:3046857, *Rattus norvegicus *(rat), gi:203804, *Oryctolagus cuniculus *(rabbit), gi:2493381, *Homo sapiens *(human), gi:28846, *Gallus gallus *(chicken), gi:211703, *Poephila guttata *(zebra finch, ovary), gi:926845, *Ovis aries *(sheep), gi:7673985.

A complete maximum likelihood analysis of the aromatase gene family was performed using the PAUP and PAML programs. The resulting tree, generated in PAUP, is shown in Figure 5, with parameters estimated using PAML. Once more, the generation of paralogs in the pig was found to have occurred after the divergence of pigs from oxen. A high K_A_/K_S _value (0.93) was again found in the divergence of the swine isoforms on the branch leading to the ancestor of the placental and embryonic enzymes following their divergence from the pig ovarian enzyme. The distribution of substitutions along this branch is consistent with altered functional constraints for the placental and embryonic enzymes compared with their extinct and extant counterparts (Tables 1 and 2) [44].

**Figure 6:**
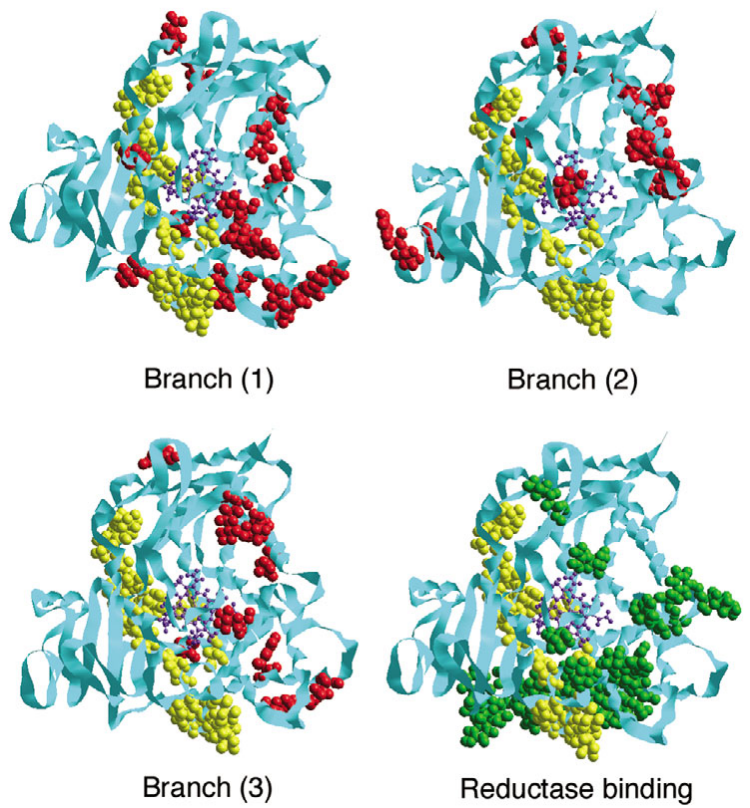
The distribution of amino acid replacements on the tertiary structure of cytochrome P450 homolog. Amino acid replacements occurring along branches highlighted in Figure 5 are shown in red. The substrate binding pocket and nicotinamide co-factor are colored yellow and purple, respectively. The sites that bind the co-reductant are highlighted in green for reference.

We correlated the episode of rapid sequence change during the emergence of the embryonic and placental paralogs with the structural biology of aromatase. A homology model of aromatase was built from progesterone 21-hydroxylase from rabbit liver (coordinates from PDB file 1DT6) [45], a homologous cytochrome P450-dependent monooxygenase. Residues undergoing replacement during the episodes represented by branches in Figure 5 (branches 1–3) are highlighted in color on the 3D model using a program in prototype with HyperChem (Figure 6).

Multiple features within the pattern of amino acid replacement were apparent. First, the sites accepting amino acid replacements in the branches with low K_A_/K_S _values (as represented by branch 2 in Figure 5) were typically scattered without any obvious pattern over the surface of the protein. This is expected for neutral drift, although an adaptive role for these replacements is not excluded by this analysis.

In contrast, the distribution of sites accepting amino acid replacements during the episode of rapid sequence evolution of branch 1 (as indicated by a relatively high K_A_/K_S _value) involving pig paralogs was not random over the protein surface. Rather, the sites are clustered near the substrate binding pocket, and in a region of the surface believed to contact the co-reductant protein, as identified by mutagenesis experiments in the homolog [46,47].

The clustering of amino acid replacements near a substrate binding site during an episode of rapid sequence evolution suggests that the substrate specificity of the protein might be changing in correlation with a change in the detailed physiological role of the protein. Recent reports suggest that the substrate and product specificities of the placental and embryonic enzymes are indeed different from those of the ovarian enzyme [23,48-50]. Further, synthesis of estrogen by the ovarian enzyme is more dependent on the structure of the co-reductant than is the placental enzyme [51]. Our *in silico *analyses rationalize these experimental observations from a structural perspective. The coupling of an evolutionary analysis to a crystallographic analysis suggests that the amino acid changes are functionally significant.

## Discussion

Today, natural history holds some of the most intellectually challenging conundrums to ever fascinate the human mind. Further, natural history offers biological chemists the opportunity to place broad biological meaning on the detailed analysis of the structure reactivity of isolated biological molecules studied in a reductionist setting. To do so, however, natural history must be connected to the physical and molecular sciences, both in subject matter and in culture.

In part to make this connection, natural historians have sought to change the research paradigm in their field to favor quantitative data directed towards the "proof" of hypotheses over "story telling". Proving hypotheses is difficult in natural history (*pace *the philosophical reality that no significant statement in empirical science can ever be said to be "proven"). The events of interest (such as the extinction of dinosaurs) are frequently distant in time, or require a passing of time (as for speciation), making them difficult to reproduce in a laboratory. The scale of the concepts involved (species, environments, planets) also does not lend these concepts to laboratory models and laboratory-controlled tests. Further, a reductionist approach, even when available, will not necessarily generate data that are relevant to the big issue that concerns the natural historian. The emphasis on data and proof has ameliorated the worst excesses of storytelling in natural history, with enormous positive impact.

Just as natural historians were purifying their field of storytelling, however, whole genome sequences began to emerge. By dramatically increasing the quantity of chemical data concerning the molecular structures of proteins, genomics changed the limiting steps in biochemical and biomedical research. No longer was the typical researcher attempting to solve an organic chemical or biotechnological question (What is the sequence of my protein? How do I express it at high levels to get the sequence?) for a protein that had been selected for functional reasons. Today, the typical researcher knows the structure of many proteins, and wishes to select one for expression and study based on a hypothesis about its potential function.

Here, the fact that any definition of function, which must make reference to fitness, requires some systems, ecological, or planetary context, makes the natural historian a natural source of hypotheses. Their full reductionist armamentarium is available in the laboratory to test and explore any hypothesis that the natural historian might provide. The biomedical researchers may like some guidance from the natural historian to narrow the broad selection, or to shorten the random walk, if only slightly.

For this purpose, the forswearing by natural historians of storytelling has come at a most inopportune time. To the modern natural historian, creating hypothesis can easily be regarded as "storytelling". They are reluctant to do so, and may criticize as atavistic colleagues who do.

This has created a vacuum in the scientific community. Very few laboratories exist that can draw upon an expertise in natural history to generate stories that create hypotheses for the researcher working in experimental biochemistry and molecular biology.

This article is designed in part to illustrate how this vacuum might be filled. Here, we do not just tell a story based on natural history, or even a story based on natural history supplemented with physiology and molecular sequence data. Rather, we show how the addition of other data, including data from X-ray crystallography, can make a story sufficiently rich that it can be viewed as being internally consistent with a wide range of independent data drawn from independent sources. This creates a hypothesis that is more than a story, even if it is less than proven.

With aromatase, the congruence of our different analyses makes a compelling suggestion that the three aromatase paralogs in pigs arose by two duplication events in the late Eocene or early Oligocene. The emergence of the aromatase paralogs corresponded approximately in time to the emergence of larger litter size in suines. This implies that the two duplication events are functionally related to the larger litter sizes. This inference is consistent with the physiological impact of estrogen synthesis by these paralogs in *Sus*. Steroid production by the porcine embryo is tightly controlled by the transient expression of aromatase and 17-hydroxylase (P450C17) between days 10 and 13 [20,21,52]. In contrast, estrogen synthesis by the equine embryo begins as early as day 6 and increases with embryo age and diameter [52]. The estrogen produced by the pig embryonic aromatase is believed to have an impact on the mobility, spacing, and implantation of the concepti [52-56]. Adequate spacing would appear to be required to manage a larger litter.

This is consistent with a structural biological analysis that correlates specific amino acid replacements with specific changes in the substrate and product specificity of the protein [57]. Interestingly, the substrate specificity of human aromatase is reported to be more similar to that displayed by the pig placental enzyme than the ovarian form [48,49]. This is an unexpected similarity given that our evolutionary analysis suggests a change in biochemical function along the fetal/placental branch in the Suidae.

It should be noted that the hypothesis is supported by the combination of data that individually would not have strength past storytelling. Thus, the K_A_/K_S _ratio of 0.93 would not, by itself, compel any particular interpretation. Its implications are greater given the relatively low K_A_/K_S _ratios of other branches of the tree. But the addition of crystallographic information, itself not compelling, makes a combination that is more compelling.

Further, this hypothesis generation itself generates discoveries that might lead to their own hypotheses. An analysis of the evolutionary branches separating pigs and humans suggests an additional episode of adaptive change. The branch leading to the ancestor of human aromatase (branch 3) has a remarkably high K_A_/K_S _ratio (13 non-synonymous and no synonymous changes; Figure 5). This *is *a K_A_/K_S _ratio greater than unity, and does (pending evaluation of its statistical significance) compel the inference of an episode of adaptive change. Intriguingly, these changes are also clustered in the same regions of the structure as those changing along branch 1 leading to the stem fetal/placental enzyme, near the substrate and co-reductant binding sites. This implies that the substrate/product specificity of the ancestral aromatase protein was not like that of either the human or the pig placental forms, but rather reflects features that arose convergently in these two species [58].

Notably, four of the sites (positions 47, 153, 219, 269) that undergo replacement during the emergence of pig placental aromatase from the last common ancestor are the same as four that arose in the emergence of the human aromatase from its last common ancestor. Of these, the amino acid replacements are identical at two sites (Thr → Met at site 153; His → Arg at site 269). The probability associated with randomly observing this pattern is extremely low (0.000021) [59]. An additional site is displaced by a single position in the sequence alignment (259/260). We hypothesize that these represent an example of adaptive parallel evolution.

It is important to point out that even an analysis this broad is likely to cover only a small part of a complicated reproductive endocrinology that must be associated with larger litter sizes. For example, the exact nature of the products produced by individual aromatases remains controversial, and may be different in laboratory studies depending on the conditions where they are studied [50,60-62]. This is especially the case with the 19-nortestosterone derivatives in Figure 1.

Further, an elegant recent study by Corbin *et al. *[23] identified 1β-hydroxytestosterone as a novel product produced by recombinant pig ovarian aromatase that is absent from the products produced by the porcine placental paralog, or by either human or bovine aromatase. This testosterone derivative binds to an androgen receptor, consistent with physiological activity. This was unknown before just this year, suggesting that more endocrine novelties remain to be discovered. Any of these may be relevant to a test of this system. For example, these hypotheses make predictions about the product specificities of the two peccary aromatases reported here.

In fact, some data suggest that uterine exposure to androgens severely decreases litter size and embryonic survival during the time of maternal recognition of pregnancy [63]. This is consistent with the hypothesis of Corbin *et al. *[50] that the evolution of the placental paralog is associated with increased efficiency of testosterone aromatization. This is also consistent with the current data, and the argument presented here.

It goes without saying that still more factors might be associated with an increase in litter size from one-two (presumed in *Diacodexis*, see Figure 4) to 12 or more in domestic swine. Most trivially, this increase might be associated with an increase in ovulation rate, and/or an adjustment in the structures and binding specificities of estrogen receptors [64].

Nevertheless, the first aromatase duplication, shared by pigs and peccaries, appears to have happened in the late Eocene (recognizing the error associated with these dates), around 35 Ma (Figure 4). This was a time of great global change, with dramatic cooling in the higher latitudes. More archaic kinds of mammals (e.g., some earlier families of perissodactyls and artiodactyls) became extinct, while many modern families (including the Suidae and Tayassuidae) became established at this time [65]. Suoids differed from other contemporaneous ungulates in their commitment to omnivory, even though a few forms, such as the modern warthog *Phacochoerus aethiopicus*, are more specialized herbivores. Perhaps the ability to bear a slightly larger litter than other artiodactyls was advantageous to them in this time of global ecological transition. However, it should be noted that larger litters usually mean altricial (i.e., relatively underdeveloped) young, a reproductive strategy apparently not available to larger, cursorial (running-adapted) ungulates, which give birth to precocial (i.e., well developed) young that are fully locomotory at birth [66].

The second aromatase duplication, with the ensuing capacity to produce multiple young, probably occurred within the family Suidae, some time during the Oligocene. The molecular data suggest dates of divergence between porcine fetal and placental aromatases as between 27–38 Ma, and the earliest known suid is of early Oligocene age [67], around 33 Ma (Figure 4). Large litters may have characterized the entire suid family. While the extant subfamily Suinae is primarily a Plio-Pleistocene radiation, during the Oligocene to Pliocene suids were exceedingly diverse taxonomically (with six other subfamilies known) as well as individually abundant as fossils [32,33,67]. In contrast, the predominantly North American tayassuids were never as diverse. It is possible that this tremendous Old-World diversity of suids, which continues to this day, is related to their capacity for the production of large litters.

This type of speculation opens questions. For example, the babirusa (an Indonesian pig) is reported to have average litters of one-two individuals [68,69]. While it is possible that litters contain three-four individuals, the occurrence is low [70]. If the common ancestor of babirusa with the African/Eurasian Suinae had a larger litter, then the babirusa must be hypothesized to represent a reversion to the more primitive condition. At present, however, relatively little is known of either the molecular biology or the natural history of babirusa. The date of divergence from modern swine is placed between 12–26 million years [71,72], while our TREx analysis using cytochrome *b *places this data at ca. 18 Ma (data not shown). Clearly, further study is warranted.

## Conclusions

The aromatase family offers an example where a combination of phylogenetic analysis, molecular evolutionary analysis, and chemical analysis set within the context of the paleontological and geological records, and supported by contemporary bioinformatics and molecular modeling tools, permits a higher order level of hypothesis generation concerning the function of proteins. Rather than simply an Enzyme Commission number (E.C. 1.14.14.1 for aromatase), a description of catalytic activity (the enzyme oxidizes testosterone), or a description of the regulatory pattern (the protein expressed between day 10 and 13), this type of analysis can generate a truly functional hypothesis: that the embryonic enzyme oxidizes testosterone as a way of managing the larger litter sizes that emerged in the Suoidea during a time of dramatic planetary cooling (ca. 35 Ma).

Such hypotheses set a higher bar, and a more useful standard, for the field of systems biology. Evolutionary theory holds that the only mechanism for obtaining functional behavior in a biological system is natural selection. Selection, based on a frequently poorly defined concept of "fitness", is determined by a context that not only includes the cell and tissue, but also the organism, the ecosystem, and a changing planet [73]. One cannot expect a collection of expression data with a mathematical model, by themselves, to provide this type of functional information unless it is set in the organismic, ecosystem, and planetary context. The historical view, of the type outlined here, becomes a critical tool for constructing this setting (Supplementary Figure [see Additional File 1]).

Humans have evidently exploited the molecular biology of larger litters to select for pigs that have truly large litters (as many as 14) following their domestication. Evidence for ancient domestication of pigs comes, *inter alia*, from a study of Indo-European languages. Proto-Indo-European (PIE) language had words for "pig" (PIE *su*-, compared with Tocharian B *suwo*, Latin *sus*, Greek *us*, Sanskrit *sukara*, Church Slavic *svinija*, Old High German *swin*, and English *sow*; also compare PIE *porko*-, with Latin *porcus*, Church Slavic *prase*, Old High German *farah*, etc. [74]), indicating that the pig has been under human domestication for at least 6000 years, enough time to have suffered a significant impact on its genotype through husbandry. We are unable, at this time, to exploit complete genome sequences of pigs or other closely related taxa to discuss the impact of domestication on aromatase, steroid receptors, amphiregulins, or other proteins that appear to be associated with uterine capacity and large litter sizes in the domesticated pig [75]. With the anticipated complete genome sequences of representatives of various mammal orders, including artiodactyls, it should be possible to extend this planetary biology approach.

## Methods

Calculations were done under the RedHat Linux 6.3 operating system on an Intel-Pentium III instrument using Blackdown's Java-SDK 1.1.8. PAML calculations were done on an IBM PC using the Unix operating system. Sequence analyses were aided by the DARWIN bioinformatics package [76]. The DARWIN package can be obtained by emailing a request to cbrg@inf.ethz.ch.

Initially, pairwise alignments were constructed for the aromatase protein sequences available in the database. An evolutionary distance in PAM units was calculated for each pair by applying the PamEstimator-package from DARWIN using an empirical log-odds matrix. From this, a preliminary evolutionary tree was built for the mammalian sequences, with branch lengths along internal nodes calculated to minimize a least-squares distance. The sequences of the ancestral genes and proteins at branch points in the tree were then reconstructed. From there, mutations (including fractional mutations) at both the DNA level and protein level were assigned to individual branches in the tree using the method of Fitch [77].

The evolutionary history of the aromatase family was then analyzed using the transition redundant exchange (TREx) metric based on an analysis of two-fold redundant codon systems [24,78]. These were obtained for each pairwise comparison of aligned aromatase genes. The number (*n*) of two-fold redundant amino acids (Cys, Asp, Glu, Phe, His, Lys, Asn, Gln, and Tyr) that are conserved in the aligned pairs was determined. The number of those amino acids that are encoded by the same codon (*c*) was determined, and the fraction (*f*_2 _= *c*/*n*) of the codons that are the same were then tabulated (Supplementary Table [see Additional File 2]). The TREx distances were calculated from *f*_2 _values using the expression *kt *= -ln((*f*_2_-E_quil_)/(1-E_quil_)), where E_quil _is the *f*_2 _value expected after a large number of nucleotide substitutions have occurred at the synonymous sites [24].

The DNA sequences for aromatase were phylogenetically analyzed using a maximum likelihood framework in PAUP 4.0* (beta 10) [79], with the following parameters: alpha value representing the gamma distribution (2.1), the transition-transversion ratio (1.6), proportion of invariable sites (0.24), and empirical base frequencies. The resulting topology of the tree mirrors those based on other molecular studies [80].

For inter-taxon analyses, families in the MasterCatalog (EraGen Biosciences, Madison WI) were identified that contained at least one representative protein from both of the taxa of interest. For these families, all inter-taxa pairs of genes were extracted, together with the pairwise protein sequence alignment. A pairwise alignment of the DNA sequences was then generated to follow the protein sequence alignment. If a family contained more than one sequence of a species belonging to one of the taxa analyzed, then those sequences were checked to determine whether they were duplicate entries into the database. If this was the case, only one of the duplicate sequences was retained in the analysis. A histogram of inter-taxa pairs was constructed, and the *f*_2 _value characteristic of orthologs determined [24]. This was used to calibrate the TREx clock using the divergence of pigs and oxen, and pigs and humans.

Codon biases were obtained from the CUTG (Codon Usage Tabulated from GenBank) made available by the Kazusa DNA Research Institute Foundation, Japan [81].

Pairwise TREx distances were used to generate lengths for the branches connecting the swine paralogs using the minimum evolution criterion in PAUP. This preliminary analysis was followed by a maximum likelihood analysis for the complete dataset using the PAML program [82]. This includes the assignment of K_A_/K_S _values to individual branches. Tests of parallel evolution were conducted using Converge [59], implementing the JTT model.

Secondary structural data based on homology modeling for aromatases were generated using the DARWIN bioinformatics package, and in agreement with previous studies [83,84]. Renderings of the three dimensional structure of the proteins were obtained using a beta version of the HyperProtein package (HyperCube, Gainesville FL, USA 32601).

## Authors' contributions

EAG performed the evolutionary, statistical and structural analyses, and prepared the manuscript. LGG cloned genes as part of his Masters work, and called the evolutionary problem to the attention of SAB. TL provided computational infrastructure. RCMS and FAS initiated the work with suid reproductive endocrinology, and supervised LGG. DRS and DAL did the initial bioinformatics analysis. CMJ provided paleontological expertise, constructed the cladogram, and helped prepare the manuscript. SAB has developed planetary biological analysis as a paradigm for generating hypotheses about the biological function of proteins, and prepared the manuscript.

## Supplementary Material

Additional File 1 Illustration of planetary biology. This figure illustrates the concepts of planetary biology as they relate to combining genomic, paleontological, chemical and ecological records to understand the history of the biosphere. Click here for file

Additional File 2 An analysis of silent nucleotide substitutions in vertebrate aromatases. The first five columns from the left indicate the index number of sequence 1 compared with sequence 2, the fraction of sites at conserved two-fold redundant coding systems that are identical (f2), the number of such sites that are conserved (c2), and the number of such sites overall (n2). The remaining columns report analogous data: for silent sites in codon systems where a change at the third nucleotide is silent only if the change is a pyrimidine-pyrimidine transition (f2y, c2y, n2y); in silent sites where a change at the third nucleotide is silent only if the change is a purine-purine transition (f2r, c2r, n2r); for the silent sites at three-fold redundant codon systems (f3, c3, n3); and for the silent sites at four-fold redundant codon systems (f4, c4, n4). Click here for file

## Figures and Tables

**Table 1:** Frequency distributions of stem pig duplication substitutions versus substitutions on all other terrestrial vertebrate branches

Terrestrial vertebrates	Non-synonymous substitutions	Synonymous substitutions	Totals
Stem pig duplicates (Branch '1' in Figure 5)	23	9	32
Remaining branches	598	1449	2047
Totals	621	1458	2079

Fisher's exact test, *P *= 0.00000094784 [44].

**Table 2:** Frequency distributions of stem pig duplication substitutions versus substitutions within the Laurasiatheria subtree

Laurasiatheria subtree	Non-synonymous substitutions	Synonymous substitutions	Totals
Stem pig duplicates (Branch '1' in Figure 5)	23	9	32
Remaining branches	232	258	490
Totals	255	267	522

Fisher's exact test, *P *= 0.0056688 [44].
