# Supplementary figures and images for: The planetary biology of cytochrome P450 aromatases
Source: BMC Biol. 2004 Aug 17;2:19. doi: 10.1186/1741-7007-2-19 (PMC515309; doi:10.1186/1741-7007-2-19)

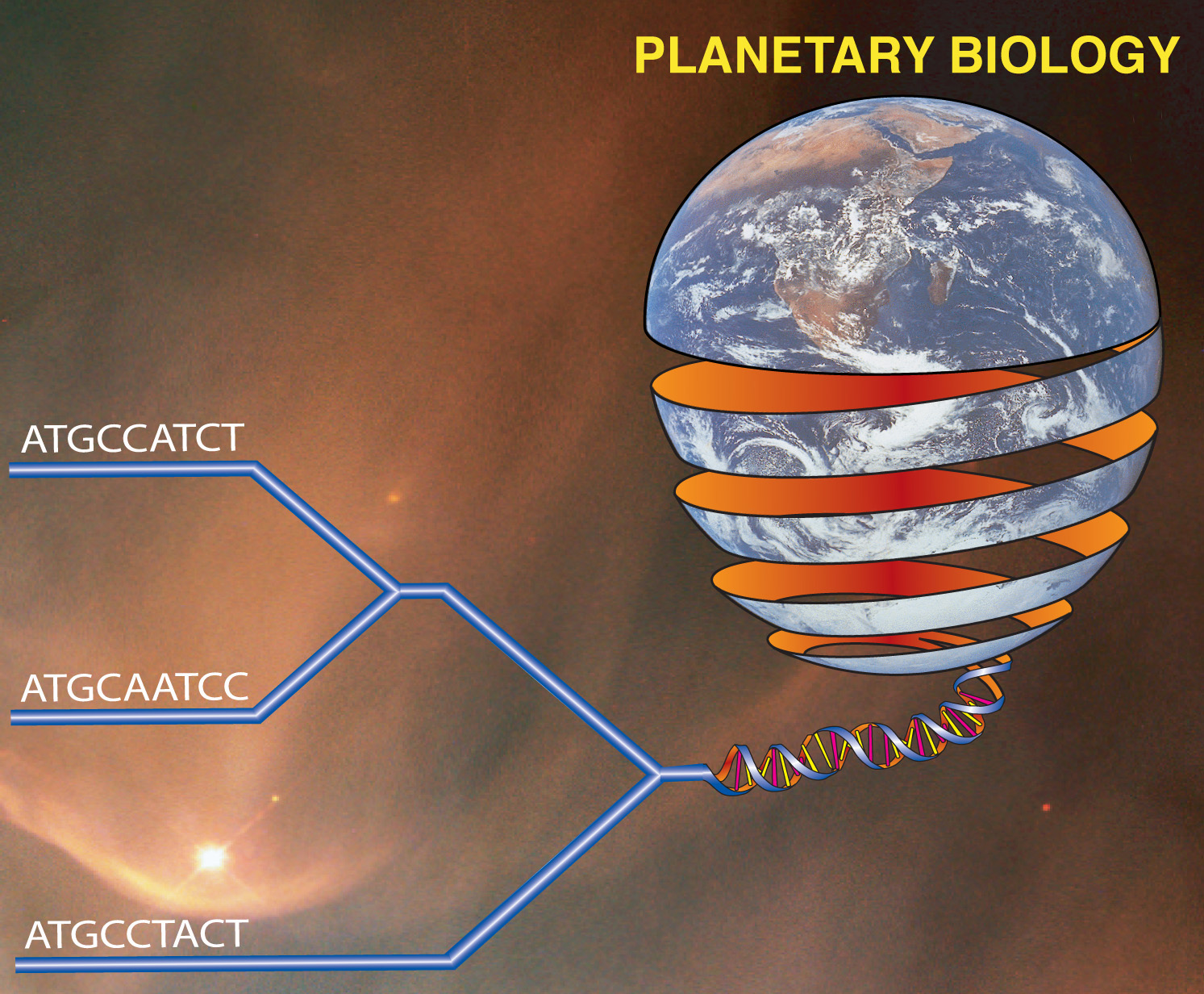

Supplement: Additional File 1 — Illustration of planetary biology. This figure illustrates the concepts of planetary biology as they relate to combining genomic, paleontological, chemical and ecological records to understand the history of the biosphere. [file 1741-7007-2-19-S1.jpeg]
