# Supplementary material for: The planetary biology of cytochrome P450 aromatases
Source: BMC Biol. 2004 Aug 17;2:19. doi: 10.1186/1741-7007-2-19 (PMC515309; doi:10.1186/1741-7007-2-19)
Supplement: Additional File 2 — An analysis of silent nucleotide substitutions in vertebrate aromatases. The first five columns from the left indicate the index number of sequence 1 compared with sequence 2, the fraction of sites at conserved two-fold redundant coding systems that are identical (f2), the number of such sites that are conserved (c2), and the number of such sites overall (n2). The remaining columns report analogous data: for silent sites in codon systems where a change at the third nucleotide is silent only if the change is a pyrimidine-pyrimidine transition (f2y, c2y, n2y); in silent sites where a change at the third nucleotide is silent only if the change is a purine-purine transition (f2r, c2r, n2r); for the silent sites at three-fold redundant codon systems (f3, c3, n3); and for the silent sites at four-fold redundant codon systems (f4, c4, n4). [file 1741-7007-2-19-S2.pdf]

Supplemental Table. An analysis of silent nucleotide substitutions in vertebrate aromatases.
The first five columns, from the left, indicate the index number of sequence 1 compared to sequence 2, the fraction of sites at conserved two fold redundant coding systems that are identical (f2), the number of such sites that are conserved (c2), and the number of such sites overall (n2). The remaining columns report analogous data, for silent sites in codon systems where a change at the third nucleotide is silent only if the change is a pyrimidine -pyrimidine transition (f2y, c2y, n2y), in silent sites where a change at the third nucleotide is silent only if the change is a purine-purine transition (f2r, c2r, n2r), the silent sites at 3 fold redundant codon systems (f3, c3, n3), and the silent sites at four fold redundant codon systems (f4, c4, n4).

1 SUS SCROFA(PIG)FETAL
2 MUS MUSCULUS(MOUSE)
3 EQUUS CABALLUS(HORSE)
4 CARASSIUS AURATUS (GOLDFISH OVARY)
5 CARASSIUS AURATUS(GOLDFISH BRAIN)
6 DANIO RERIO(ZEBRAFISH)
7 SUS SCROFA(PIG)PLACENTAL
8 ORYZIAS LATIPES(MEDAKA)
9 SUS SCROFA(PIG)OVARY
10 ORYCTOLAGUS CUNICULUS(RABBIT)
11 ICTALURUS PUNCTATUS(CHANNEL CATFISH)
12 TAENIOPYGIA GUTTATA(ZEBRA FINCH)
13 ONCORHYNCHUS MYKISS (RAINBOWTROUT)
14 BOS TAURUS(OX)
15 HOMO SAPIENS(HUMAN)
16 GALLUS GALLUS(CHICKEN)
17 RATTUS NORVEGICUS(RAT)
18 OVIS ARIES(SHEEP)

seq1	seq2	f2	c2	n2	f2y	c2y	n2y	f2r	c2r	n2r	f3	c3	n3	f4	c4	n4
1	2	0.703	97	138	0.700	56	80	0.707	41	58	0.714	20	28	0.650	65	100
1	3	0.837	123	147	0.845	71	84	0.825	52	63	0.792	19	24	0.786	77	98
1	4	0.625	55	88	0.642	34	53	0.600	21	35	0.312	5	16	0.354	23	65
1	5	0.569	58	102	0.638	37	58	0.477	21	44	0.333	6	18	0.333	23	69
1	6	0.600	54	90	0.685	37	54	0.472	17	36	0.176	3	17	0.381	24	63
1	7	0.920	161	175	0.908	89	98	0.935	72	77	0.938	30	32	0.952	118	124

1	8	0.546	53	97	0.603	35	58	0.462	18	39	0.471	8	17	0.353	24	68
1	9	0.929	156	168	0.936	88	94	0.919	68	74	0.966	28	29	0.947	107	113
1	10	0.727	112	154	0.678	59	87	0.791	53	67	0.710	22	31	0.733	77	105
1	11	0.565	48	85	0.529	27	51	0.618	21	34	0.412	7	17	0.388	26	67
1	12	0.643	83	129	0.615	48	78	0.686	35	51	0.556	15	27	0.416	37	89
1	13	0.568	50	88	0.630	34	54	0.471	16	34	0.133	2	15	0.388	26	67
1	14	0.842	139	165	0.809	76	94	0.887	63	71	0.839	26	31	0.841	95	113
1	15	0.808	126	156	0.778	70	90	0.848	56	66	0.724	21	29	0.755	77	102
1	16	0.608	76	125	0.639	46	72	0.566	30	53	0.615	16	26	0.376	32	85
1	17	0.689	93	135	0.620	49	79	0.786	44	56	0.667	20	30	0.694	68	98
1	18	0.846	143	169	0.811	77	95	0.892	66	74	0.871	27	31	0.825	94	114
2	3	0.739	105	142	0.726	61	84	0.759	44	58	0.773	17	22	0.642	61	95
2	4	0.483	42	87	0.463	25	54	0.515	17	33	0.368	7	19	0.270	17	63
2	5	0.527	49	93	0.571	32	56	0.459	17	37	0.238	5	21	0.338	24	71
2	6	0.511	45	88	0.545	30	55	0.455	15	33	0.400	8	20	0.393	24	61
2	7	0.706	96	136	0.696	55	79	0.719	41	57	0.741	20	27	0.643	63	98
2	8	0.674	62	92	0.638	37	58	0.735	25	34	0.600	12	20	0.250	17	68
2	9	0.711	101	142	0.702	59	84	0.724	42	58	0.708	17	24	0.670	65	97
2	10	0.736	106	144	0.744	64	86	0.724	42	58	0.576	19	33	0.600	63	105
2	11	0.615	56	91	0.596	34	57	0.647	22	34	0.500	10	20	0.339	21	62
2	12	0.569	74	130	0.550	44	80	0.600	30	50	0.556	15	27	0.367	33	90
2	13	0.671	53	79	0.700	35	50	0.621	18	29	0.389	7	18	0.358	24	67
2	14	0.719	100	139	0.706	60	85	0.741	40	54	0.655	19	29	0.624	63	101
2	15	0.748	107	143	0.765	65	85	0.724	42	58	0.656	21	32	0.687	68	99
2	16	0.584	73	125	0.613	46	75	0.540	27	50	0.462	12	26	0.368	32	87
2	17	0.871	149	171	0.853	87	102	0.899	62	69	0.838	31	37	0.817	94	115
2	18	0.741	106	143	0.733	63	86	0.754	43	57	0.714	20	28	0.596	59	99
3	4	0.549	50	91	0.491	27	55	0.639	23	36	0.545	6	11	0.333	22	66
3	5	0.545	54	99	0.556	30	54	0.533	24	45	0.286	4	14	0.303	20	66
3	6	0.565	52	92	0.571	32	56	0.556	20	36	0.417	5	12	0.365	23	63
3	7	0.819	118	144	0.817	67	82	0.823	51	62	0.773	17	22	0.755	74	98
3	8	0.562	54	96	0.569	33	58	0.553	21	38	0.500	7	14	0.314	22	70
3	9	0.836	122	146	0.855	71	83	0.810	51	63	0.727	16	22	0.768	73	95
3	10	0.748	110	147	0.721	62	86	0.787	48	61	0.708	17	24	0.700	70	100

3	11	0.506	45	89	0.455	25	55	0.588	20	34	0.538	7	13	0.373	25	67
3	12	0.620	85	137	0.612	52	85	0.635	33	52	0.545	12	22	0.356	31	87
3	13	0.560	51	91	0.600	33	55	0.500	18	36	0.500	6	12	0.456	31	68
3	14	0.813	122	150	0.809	72	89	0.820	50	61	0.783	18	23	0.733	74	101
3	15	0.819	122	149	0.824	70	85	0.812	52	64	0.720	18	25	0.762	77	101
3	16	0.646	84	130	0.667	52	78	0.615	32	52	0.545	12	22	0.306	26	85
3	17	0.741	103	139	0.675	56	83	0.839	47	56	0.792	19	24	0.621	59	95
3	18	0.810	124	153	0.798	71	89	0.828	53	64	0.783	18	23	0.733	74	101
4	5	0.649	72	111	0.652	43	66	0.644	29	45	0.650	13	20	0.443	35	79
4	6	0.810	132	163	0.828	82	99	0.781	50	64	0.714	20	28	0.686	70	102
4	7	0.535	46	86	0.529	27	51	0.543	19	35	0.294	5	17	0.323	21	65
4	8	0.602	74	123	0.625	45	72	0.569	29	51	0.571	12	21	0.341	30	88
4	9	0.604	55	91	0.603	35	58	0.606	20	33	0.250	4	16	0.313	21	67
4	10	0.544	49	90	0.561	32	57	0.515	17	33	0.200	4	20	0.329	23	70
4	11	0.677	86	127	0.688	55	80	0.660	31	47	0.652	15	23	0.427	38	89
4	12	0.589	56	95	0.554	31	56	0.641	25	39	0.421	8	19	0.412	28	68
4	13	0.635	73	115	0.638	44	69	0.630	29	46	0.667	12	18	0.410	34	83
4	14	0.567	51	90	0.544	31	57	0.606	20	33	0.412	7	17	0.313	21	67
4	15	0.540	47	87	0.537	29	54	0.545	18	33	0.450	9	20	0.354	23	65
4	16	0.581	54	93	0.556	30	54	0.615	24	39	0.474	9	19	0.377	26	69
4	17	0.523	45	86	0.528	28	53	0.515	17	33	0.333	6	18	0.323	20	62
4	18	0.565	52	92	0.561	32	57	0.571	20	35	0.353	6	17	0.299	20	67
5	6	0.602	68	113	0.574	39	68	0.644	29	45	0.500	10	20	0.494	38	77
5	7	0.520	52	100	0.527	29	55	0.511	23	45	0.200	4	20	0.304	21	69
5	8	0.583	63	108	0.540	34	63	0.644	29	45	0.500	9	18	0.375	30	80
5	9	0.560	56	100	0.600	36	60	0.500	20	40	0.333	6	18	0.319	22	69
5	10	0.525	52	99	0.552	32	58	0.488	20	41	0.409	9	22	0.296	21	71
5	11	0.590	62	105	0.585	38	65	0.600	24	40	0.667	12	18	0.436	34	78
5	12	0.535	54	101	0.569	33	58	0.488	21	43	0.474	9	19	0.333	23	69
5	13	0.640	71	111	0.642	43	67	0.636	28	44	0.467	7	15	0.467	35	75
5	14	0.515	50	97	0.554	31	56	0.463	19	41	0.353	6	17	0.324	22	68
5	15	0.525	52	99	0.544	31	57	0.500	21	42	0.364	8	22	0.364	24	66
5	16	0.545	54	99	0.544	31	57	0.548	23	42	0.421	8	19	0.296	21	71
5	17	0.516	48	93	0.571	32	56	0.432	16	37	0.250	5	20	0.366	26	71

5	18	0.505	50	99	0.554	31	56	0.442	19	43	0.353	6	17	0.319	22	69
6	7	0.580	51	88	0.596	31	52	0.556	20	36	0.278	5	18	0.365	23	63
6	8	0.685	85	124	0.712	52	73	0.647	33	51	0.524	11	21	0.356	31	87
6	9	0.602	56	93	0.678	40	59	0.471	16	34	0.235	4	17	0.375	24	64
6	10	0.620	57	92	0.672	39	58	0.529	18	34	0.286	6	21	0.457	32	70
6	11	0.674	87	129	0.688	55	80	0.653	32	49	0.609	14	23	0.494	43	87
6	12	0.617	58	94	0.649	37	57	0.568	21	37	0.400	8	20	0.394	26	66
6	13	0.625	75	120	0.606	43	71	0.653	32	49	0.706	12	17	0.456	36	79
6	14	0.554	51	92	0.569	33	58	0.529	18	34	0.444	8	18	0.339	21	62
6	15	0.602	53	88	0.648	35	54	0.529	18	34	0.381	8	21	0.367	22	60
6	16	0.630	58	92	0.655	36	55	0.595	22	37	0.300	6	20	0.397	27	68
6	17	0.494	43	87	0.519	28	54	0.455	15	33	0.421	8	19	0.459	28	61
6	18	0.553	52	94	0.586	34	58	0.500	18	36	0.389	7	18	0.323	20	62
7	8	0.556	55	99	0.552	32	58	0.561	23	41	0.562	9	16	0.343	23	67
7	9	0.910	151	166	0.903	84	93	0.918	67	73	0.929	26	28	0.928	103	111
7	10	0.765	114	149	0.687	57	83	0.864	57	66	0.767	23	30	0.733	77	105
7	11	0.518	44	85	0.480	24	50	0.571	20	35	0.500	9	18	0.379	25	66
7	12	0.643	83	129	0.649	50	77	0.635	33	52	0.538	14	26	0.425	37	87
7	13	0.489	43	88	0.528	28	53	0.429	15	35	0.143	2	14	0.369	24	65
7	14	0.848	134	158	0.798	71	89	0.913	63	69	0.900	27	30	0.793	88	111
7	15	0.810	124	153	0.759	66	87	0.879	58	66	0.767	23	30	0.743	75	101
7	16	0.603	76	126	0.681	49	72	0.500	27	54	0.542	13	24	0.381	32	84
7	17	0.727	96	132	0.636	49	77	0.855	47	55	0.655	19	29	0.667	64	96
7	18	0.833	135	162	0.789	71	90	0.889	64	72	0.931	27	29	0.795	89	112
8	9	0.592	58	98	0.650	39	60	0.500	19	38	0.529	9	17	0.319	22	69
8	10	0.688	66	96	0.717	43	60	0.639	23	36	0.474	9	19	0.315	23	73
8	11	0.647	77	119	0.662	47	71	0.625	30	48	0.550	11	20	0.424	36	85
8	12	0.480	47	98	0.541	33	61	0.378	14	37	0.389	7	18	0.417	30	72
8	13	0.757	115	152	0.719	64	89	0.810	51	63	0.577	15	26	0.625	60	96
8	14	0.573	55	96	0.542	32	59	0.622	23	37	0.588	10	17	0.324	22	68
8	15	0.642	61	95	0.638	37	58	0.649	24	37	0.714	15	21	0.318	21	66
8	16	0.500	47	94	0.552	32	58	0.417	15	36	0.263	5	19	0.338	24	71
8	17	0.663	61	92	0.684	39	57	0.629	22	35	0.526	10	19	0.235	16	68
8	18	0.582	57	98	0.559	33	59	0.615	24	39	0.667	12	18	0.348	24	69

9	10	0.752	115	153	0.727	64	88	0.785	51	65	0.833	25	30	0.750	78	104
9	11	0.581	50	86	0.566	30	53	0.606	20	33	0.500	8	16	0.388	26	67
9	12	0.652	86	132	0.684	54	79	0.604	32	53	0.593	16	27	0.386	34	88
9	13	0.540	47	87	0.600	33	55	0.438	14	32	0.143	2	14	0.397	27	68
9	14	0.842	139	165	0.821	78	95	0.871	61	70	0.893	25	28	0.827	91	110
9	15	0.860	135	157	0.846	77	91	0.879	58	66	0.786	22	28	0.730	73	100
9	16	0.623	81	130	0.693	52	75	0.527	29	55	0.538	14	26	0.365	31	85
9	17	0.721	98	136	0.637	51	80	0.839	47	56	0.654	17	26	0.702	66	94
9	18	0.839	141	168	0.821	78	95	0.863	63	73	0.931	27	29	0.811	90	111
10	11	0.621	54	87	0.600	33	55	0.656	21	32	0.500	10	20	0.443	31	70
10	12	0.610	83	136	0.600	51	85	0.627	32	51	0.469	15	32	0.350	35	100
10	13	0.598	52	87	0.618	34	55	0.562	18	32	0.118	2	17	0.478	33	69
10	14	0.760	117	154	0.700	63	90	0.844	54	64	0.844	27	32	0.661	72	109
10	15	0.813	126	155	0.767	69	90	0.877	57	65	0.824	28	34	0.745	82	110
10	16	0.579	77	133	0.595	47	79	0.556	30	54	0.367	11	30	0.316	30	95
10	17	0.764	107	140	0.741	63	85	0.800	44	55	0.576	19	33	0.631	65	103
10	18	0.731	114	156	0.656	59	90	0.833	55	66	0.812	26	32	0.667	72	108
11	12	0.578	52	90	0.582	32	55	0.571	20	35	0.444	8	18	0.386	27	70
11	13	0.652	73	112	0.681	47	69	0.605	26	43	0.500	8	16	0.469	38	81
11	14	0.558	48	86	0.528	28	53	0.606	20	33	0.667	12	18	0.403	27	67
11	15	0.565	48	85	0.490	25	51	0.676	23	34	0.600	12	20	0.431	28	65
11	16	0.562	50	89	0.566	30	53	0.556	20	36	0.222	4	18	0.314	22	70
11	17	0.644	58	90	0.636	35	55	0.657	23	35	0.421	8	19	0.381	24	63
11	18	0.557	49	88	0.509	27	53	0.629	22	35	0.647	11	17	0.424	28	66
12	13	0.538	50	93	0.569	33	58	0.486	17	35	0.400	6	15	0.478	33	69
12	14	0.659	87	132	0.610	50	82	0.740	37	50	0.481	13	27	0.411	37	90
12	15	0.615	83	135	0.646	53	82	0.566	30	53	0.500	14	28	0.385	35	91
12	16	0.771	131	170	0.780	78	100	0.757	53	70	0.632	24	38	0.602	68	113
12	17	0.580	76	131	0.550	44	80	0.627	32	51	0.630	17	27	0.352	31	88
12	18	0.664	89	134	0.622	51	82	0.731	38	52	0.500	14	28	0.411	37	90
13	14	0.619	52	84	0.654	34	52	0.562	18	32	0.267	4	15	0.409	27	66
13	15	0.643	54	84	0.654	34	52	0.625	20	32	0.353	6	17	0.469	30	64
13	16	0.533	48	90	0.518	29	56	0.559	19	34	0.375	6	16	0.357	25	70
13	17	0.582	46	79	0.640	32	50	0.483	14	29	0.412	7	17	0.379	25	66

13	18	0.624	53	85	0.673	35	52	0.545	18	33	0.250	4	16	0.403	27	67
14	15	0.824	131	159	0.793	73	92	0.866	58	67	0.933	28	30	0.673	72	107
14	16	0.638	81	127	0.653	49	75	0.615	32	52	0.462	12	26	0.391	34	87
14	17	0.715	98	137	0.643	54	84	0.830	44	53	0.613	19	31	0.602	59	98
14	18	0.962	175	182	0.954	103	108	0.973	72	74	0.973	36	37	0.984	122	124
15	16	0.600	78	130	0.640	48	75	0.545	30	55	0.423	11	26	0.391	34	87
15	17	0.754	107	142	0.694	59	85	0.842	48	57	0.581	18	31	0.653	62	95
15	18	0.839	135	161	0.817	76	93	0.868	59	68	0.897	26	29	0.676	71	105
16	17	0.591	75	127	0.605	46	76	0.569	29	51	0.462	12	26	0.333	28	84
16	18	0.636	82	129	0.653	49	75	0.611	33	54	0.444	12	27	0.384	33	86
17	18	0.716	101	141	0.647	55	85	0.821	46	56	0.633	19	30	0.577	56	97
